# NLRP3 Triggers Attenuate Lipocalin-2 Expression Independent with Inflammasome Activation

**DOI:** 10.3390/cells10071660

**Published:** 2021-07-02

**Authors:** Huijeong Ahn, Gilyoung Lee, Jeongeun Kim, Jeongho Park, Seung Goo Kang, Sung-Il Yoon, Eunsong Lee, Geun-Shik Lee

**Affiliations:** 1 College of Veterinary Medicine and Institute of Veterinary Science, Kangwon National University, Chuncheon, Gangwon 24341, Korea; balloon1981@naver.com (H.A.); lky123001@gmail.com (G.L.); rlawjddms45@naver.com (J.K.); jhp@kangwon.ac.kr (J.P.); eslee@kangwon.ac.kr (E.L.); 2 Division of Biomedical Convergence, College of Biomedical Science, Kangwon National University, Chuncheon, Gangwon 24341, Korea; sgkang@kangwon.ac.kr (S.G.K.); sungil@kangwon.ac.kr (S.-I.Y.)

**Keywords:** lipocalin-2, NLRP3 inflammasome, nigericin, macrophages

## Abstract

Lipocalin-2 (LCN2), a small secretory glycoprotein, is upregulated by toll-like receptor (TLR) signaling in various cells and tissues. LCN2 inhibits bacterial growth by iron sequestration and regulates the innate immune system. Inflammasome activates the inflammatory caspases leading to pyroptosis and cytokine maturation. This study examined the effects of inflammasome activation on LCN2 secretion in response to TLR signaling. The triggers of NLRP3 inflammasome activation attenuated LCN2 secretion while it induced interleukin-1β in mouse macrophages. In mice, NLRP3 inflammasome activation inhibited TLR-mediated LCN2 secretion. The inhibition of NLRP3 triggers on LCN2 secretion was caused by the inhibited transcription and translation of LCN2. At the same time, no changes in the other cytokines and IκBζ, a well-known transcriptional factor of *Lcn2* transcription, were observed. Overall, NLRP3 triggers are a regulator of LCN2 expression suggesting a new linkage of inflammasome activation and LCN2 secretion in the innate immunity.

## 1. Introduction

Lipocalins, small secreted proteins (160 to 180 amino acids), have a high affinity to lipophilic molecules and soluble macromolecules, such as neutrophil gelatinase [1]. Lipocalins were first defined as a transport protein, but several lipocalins, α1-microglobulin, glycodelin, and lipocalin-2, have been reported to be involved in a range of biological functions, such as cell growth and metabolism, and immune responses [1]. Lipocalin-2 (LCN2), also designated as neutrophil gelatinase-associated lipocalin (NGAL) or 24p3, is a secretory glycoprotein (25 kDa) identified from neutrophil granules and is known as an acute-phase protein of the liver [2]. LCN2 is upregulated by interleukin (IL)-1β, IL-22, and toll-like receptor (TLR) ligands in diverse cells and tissues, such as neutrophils, monocytes, macrophages, adipocytes, kidney, prostate, epithelium of the respiratory system and intestines [2,3]. Thus, LCN2 has been reported to be a useful biomarker of early inflammation and tissue injury [2].

LCN2 showed two significant functions in the immune response: limitation of bacterial growth and regulation of innate immune cells [1,4]. Because bacteria need iron for growth and metabolism, the invading pathogens use iron-binding proteins (i.e., siderophore) to acquire the host iron [1,5]. The host secretes LCN2 through TLR signaling in response to the pathogen [4,5]. LCN2 binds to siderophore and prevents the pathogen from acquiring iron, limiting bacterial growth [5]. Therefore, the bactericidal activity of LCN2-deficient immune cells is attenuated, and LCN2-gene knockout mice are prone to infection and sepsis [2,4]. In addition, LCN2 ameliorates intestinal inflammation by changing the composition of the gut microbiota [2]. LCN2 also regulates host cells directly via its receptor [2]. Animal models of type 2 diabetes and nonalcoholic steatohepatitis showed increased LCN2 secretion, which recruits inflammatory cells and induces proinflammatory cytokines, suggesting that LCN2 induces sterile inflammation [2]. On the other hand, obesity-mediated inflammation of LCN2 gene-depleted mice elicits more expression of proinflammatory markers (e.g., IL-1β, IL-6, iNOS, and MCP-1) than WT mice [6]. In addition, the pretreatment of recombinant LCN2 in macrophages attenuated lipopolysaccharide (LPS)-mediated proinflammatory expression [6].

Inflammasome, intracellular multi-protein complexes, is assembled by endogenous and pathogenic danger molecules [7,8]. Inflammasomes, which are composed of a sensor protein (e.g., NLRP3, NLRC4, and AIM2), an adaptor protein (e.g., ASC), and pro-caspase-1, are classified by a sensor protein, which recognizes the cytoplasmic danger signals [9]. NLRP3 inflammasomes are assembled by adenosine triphosphate (ATP), nigericin (NG), and monosodium urate (MSU) crystals inducing potassium efflux [9]. The potassium efflux is blocked by extracellular high potassium chloride, and the assembly of NLRP3 inflammasome is also inhibited by MCC950 [9,10]. NLRC4 or AIM2 proteins directly bind with intracellular flagellin and dsDNA leading to the assembly of inflammasome [7,8]. The effector of inflammasome activation, caspase-1, is blocked by caspase inhibitors (e.g., Z-VAD-FMK) [11]. The activation of inflammasome induces inflammatory cell death (i.e., pyroptosis) and maturate cytokines (i.e., IL-1β) via the inflammatory caspases, caspase-1 and -11 [7,8].

IL-1β upregulates LCN2 in the respiratory epithelium and keratinocytes, but TNFα does not [12]. In contrast, LCN2 activates NLRP3 inflammasome through high mobility group box 1 (HMGB1) secretion and mitochondrial dysfunction in cardiomyocytes [13]. HMGB1 induces the priming step of inflammasome activation, and mitochondrial reactive oxygen species and DNA stimulates the activation step [13,14]. These data suggest that LCN2 secretion and inflammasome are tightly regulated by each other. Thus, this study first examined the effect of inflammasome activation on LCN2 secretion in macrophages. The role of inflammasome triggers on LCN2 production was examined further in a macrophage-like cell line and mouse. The physiological meaning of the regulation of LCN2 by NLRP3 triggers was also speculated.

## 2. Materials and Methods

### 2.1. Cell Culturing and Treatment

**Figure 1:**
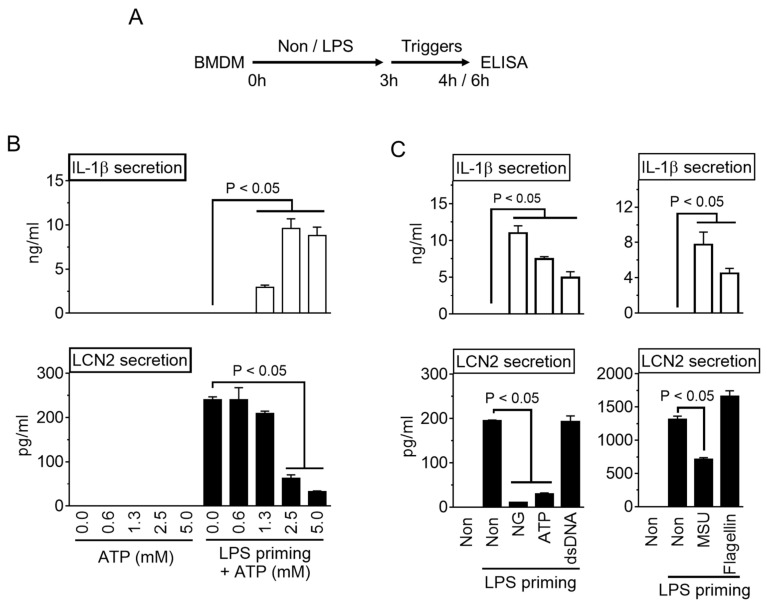
Effect of inflammasome activation on LCN2 secretion. (**A**) Schematic diagram of the experimental process. BMDM were primed with LPS for 3 h, and treated with a specific inflammasome trigger for 1 h or 3 h. The cellular supernatant was subjected to ELISA to measure the LCN2 or IL-1β levels. (**B**) BMDM were primed with/without LPS for 3 h, and then treated with increasing dosages of ATP for 1 h. The secretion of IL-1β and LCN2 were measured by ELISA. (**C**) LPS-primed BMDM were treated with the triggers of NLRP3 (NG, ATP, and MSU), AIM2 (dsDNA), or NLRC4 (flagellin) inflammasome. The IL-1β and LCN2 secretion were observed by ELISA. The bar graph presents the mean ± standard deviation (SD) with at least two independent experiments.

**Figure 2:**
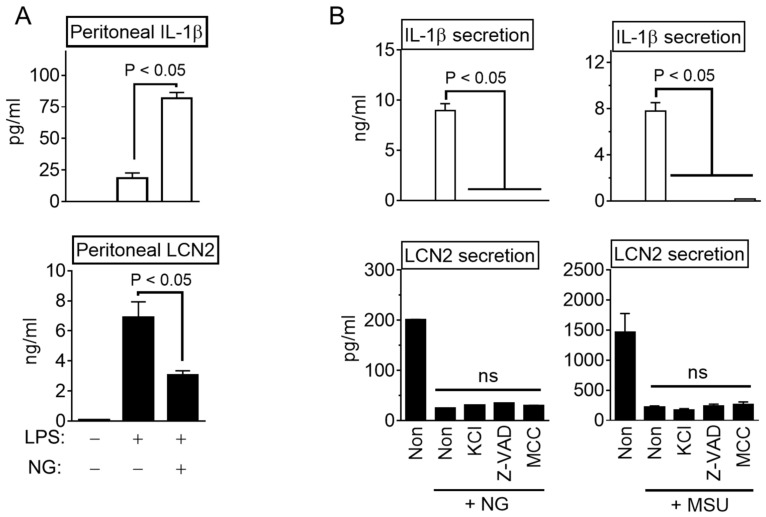
LCN2 secretion and the effect of inflammasome inhibitors on LCN2 secretion. (**A**) Mice (six mice/group) were ip injected with LPS 5 h before the NG treatment and then sacrificed 1 h after NG injection. Peritoneal lavages subjected to IL-1β and LCN2 ELISA. (**B**) LPS-primed BMDM were treated with NG or MSU, an NLRP3 trigger, and the secretion of IL-1β and LCN2 in the cellular supernatants were analyzed by ELISA. The bar graph indicates the mean ± SD with at least two independent experiments. ns, not significant.

**Figure 3:**
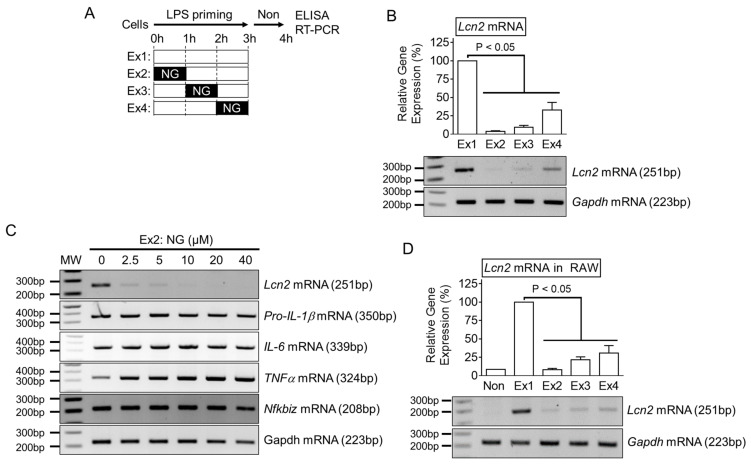
Effect of NG on *Lcn2* transcription. (**A**) Schematic diagram of the experimental process. (**B**) BMDM were treated with LPS for three h in the presence of NG, as indicated in panel A. *Lcn2* mRNA expression was analyzed by RT-PCR, and the band intensity is represented as a bar graph. (**C**) BMDM were treated with LPS and various dosages of NG, as indicated in Ex2 of panel A. Gene expression was analyzed by RT-PCR. (**D**) Raw 264.7 cells were treated with LPS and NG, as shown in panel A. *Lcn2* gene expression was assayed by RT-PCR, and the band density is presented as the bar graphs. The bar graph shows the mean ± SD of at least two independent experiments.

Progenitor cells from the bone marrow were isolated from the femur and tibia of C57BL/6 mice (Nara Biotech, Seoul, Korea) and differentiated into macrophages for seven days in RPMI 1640 medium (Welgene Inc., Gyeongsan-si, Korea) containing 10% fetal bovine serum (FBS, VWR International, Wayne, PA, USA), antibiotics (CA005, GenDEPOT, Inc., Barker, TX, USA), and 30% of L929 cell-conditioned media containing macrophage colony-stimulating factor. The Raw 264.7 cell line (#40071, Korean Cell Line Bank, Seoul, Korea) was cultivated in DMEM media (Welgene Inc.) containing 10% FBS and antibiotics. All cells were incubated at 37 °C in a 5% CO_2_ atmosphere. To activate inflammasome (Figure 1A), bone marrow-derived macrophages (BMDM) were plated into a culturing plate (1.0 × 10^6^ cells per well in 12-well-plate, SPL Life Science Co., Seoul, Korea), and then primed with lipopolysaccharide (LPS, L4130, Sigma–Aldrich Co., MO, USA) for 3 h in RPMI 1649 containing 10% FBS and antibiotics. After LPS priming, the cells were subjected to RPMI 1649 medium containing inflammasome triggers as follows: adenosine triphosphate (ATP, Invitrogen, CA, USA) for 1 h; nigericin (NG, 40 μM; 4312, Tocris Bioscience, Bristol, UK) for 1 h; dsDNA (1 μg/mL) with jetPRIME^TM^ (2 μL/mL, Polyplus-transfection Inc., Illkirch, France) for 1 h [15]; monosodium urate crystals (MSU, 400 μg/mL; U2875, Sigma–Aldrich Co.) which were prepared according to a previous study [16] for 3 h; flagellin (500 ng/mL, InvivoGen) with Lipofectamine 2000 (10 μL/mL, Invitrogen, Carlsbad, CA, USA) for 3 h. For the inflammasome inhibitor experiment (Figure 2B), LPS-primed BMDM were activated by NLRP3 inflammasome and NG or MSU in the presence of KCl (50 mM, Biosesang, Seoul, Korea), Z-VAD-FMK (10 μL/mL, R&D Systems, Minneapolis, MN, USA), or MCC950 (200 nM, Invivogen). For transcripts analysis, BMDM or Raw 264.7 cells were seeded onto a 6-well plate (2.0 × 10^6^ cells per well, SPL Life Science Co.), and treated with LPS (10 ng/mL) for 3 h with NG (10 μM), as indicated in Figure 3A.

### 2.2. Animal Study

Female mice (C57BL/6, eight-week-old, Nara Biotech) were maintained at 18 to 24 °C under a 12 h light/dark cycle and supplied with standard chow diet and tap water ad libitum. The mice (*n* = 6 per group, total *n* = 18) were injected intraperitoneally (ip) with LPS (100 μg/mouse) 5 h before NG (60 μg/mouse) ip administration [17]. The mice were sacrificed by CO_2_ inhalation 1 h after the NG treatment, and the peritoneal lavages were harvested by washing with 5 mL of phosphate-buffered saline [15]. The animal experiments were conducted under the National Institutes of Health Guide for the Care and Using of Laboratory Animals and approved by the Institutional Animal Care and Use Committee of Kangwon National University (approval no. KW-200210-2).

### 2.3. Detection of LCN2, IL-1β, TNFα, IL-6 Using ELISA

The cellular supernatants were harvested from 12-well plates. The cells were lysed with a mild lysis buffer (150 mM NaCl, 1% Triton X-100, 50 mM Tri-base, pH 8.0) containing the proteinase inhibitors (Halt proteinase inhibitor cocktail, ThermoFisher Scientific, Waltham, MA, USA). The cellular lysate (Lys) was collected after centrifugation. Mouse LCN2, IL-1β, TNFα, or IL-6 secretion in the cellular supernatant and lysate, and mouse peritoneal lavages were analyzed using an ELISA Kit (DY1857, DY410, DY406, or DY401 R&D Systems) and a microplate spectrophotometer (Synergy™ H1 Hybrid Multi-Mode Reader, BioTek, Winooski, VT, USA).

### 2.4. Western Blot Analysis

Lys were separated by SDS-PAGE (10%) and transferred to a polyvinylidene difluoride membrane (PVDF; GE Healthcare Bio-Science, Pittsburgh, PA, USA). The membrane was incubated with the primary antibodies against anti-mouse LCN2 antibody (AF1857, R&D Systems, Minneapolis, MN, USA), anti-mouse IL-1β antibody (AF-401-NA, R&D Systems), or anti-Actin antibody (sc-1615, Santa Cruz Biotechnology, Dallas, TA, USA) overnight at 4 °C. The PVDF was further probed with 2nd anti-sera conjugated with horseradish peroxidase (donkey anti-goat IgG for LCN2 and IL-1β anti-sera, ab6885, Abcam, Cambridge, UK) and visualized using an enhanced chemiluminescence solution (WESTSAVER STAR, AbFrontier, Seoul, Korea) and a chemiluminescent system (EZ-Capture II, ATTO Technology, Tokyo, Japan).

### 2.5. RNA Extraction and Reverse Transcription-Polymerase Chain Reaction (RT-PCR)

The total RNA of BMDM and Raw 264.7 cells was extracted by NucleoZOL (MACHEREY-NAGEL GmbH & Co. KG, Postfach, Düren, Germany) and reverse-transcribed into first-strand complementary DNA (cDNA) using a random primer (9-mer, Invitrogen) and M-MLV reverse transcriptase (Enzynomics Co., Daejeon, Korea) [18]. The transcription was amplified using a SimpliAmp Thermal Cycler (Thermo Fisher Scientific, Waltham, MA, USA) and nTaq polymerase (Enzynomics). The PCR products were visualized by agarose gel electrophoresis and ethidium bromide staining. The band intensity was analyzed using a CS Analyzer (Ver. 3, ATTO Technology). Supplementary Table S1 shows the primer sequences.

### 2.6. Statistical Analyses

Statistical analyses were carried out using GraphPad Prism 6 (GraphPad Software, San Diego, CA, USA): Mann-Whitney test for the two groups or one-way ANOVA (Tukey’s multiple comparisons test) for multiple groups. The *p*-value is presented in the figure.

## 3. Results

### 3.1. NLRP3 Inflammasome Activation Attenuate LCN2 Secretion

Mouse BMDM were primed with LPS, and NLRP3 inflammasome was then triggered by ATP, as indicated in Figure 1A. As expected, LPS-primed BMDM provoked IL-1β secretion, an indicator of inflammasome activation, depending on the increasing dosages of ATP (Figure 1B). The same cellular supernatant was analyzed to determine the effects of inflammasome activation on LCN2 secretion (Figure 1C). LPS priming induced LCN2 release from BMDM. The ATP treatment inhibited the releases of LCN2 in a dose-dependent manner. Thus, inflammasome activation suppressed LCN2 secretion while eliciting IL-1β maturation. The inhibitory property of the inflammasome activation on LCN2 secretion was further confirmed using several inflammasome triggers. Similar to Figure 1A, BMDM were primed with LPS and treated with NLRP3 triggers (NG, ATP, and MSU), AIM2 trigger (dsDNA), and NLRC4 trigger (flagellin). As a result, all inflammasome triggers induced IL-1β secretion from LPS-primed BMDM. On the other hand, LPS-mediated LCN2 secretion was attenuated by NLRP3 triggers but not by AIM2 and NLRC4 triggers. Overall, NLRP3 inflammasome activation selectively suppresses LCN2 release.

### 3.2. NLRP3 Triggers Inhibit LCN2 Secretion Independent Inflammasome Activation

The inhibitory property of NLRP3 inflammasome activation on the reduction in LCN2 secretion in animals was confirmed. The mice were injected with LPS and then treated with NG to stimulate IL-1β maturation through NLRP3 inflammasome activation. As shown in Figure 2A, peritoneal IL-1β releases were simulated by the NG treatment, but LPS-mediated LCN2 secretion decreased in the NG-injected mice. Therefore, the regulatory efficacy of the NLRP3 trigger on LCN2 secretion was reproduced in mice. Next, several inhibitors, such as the inhibitor of potassium efflux (high KCl solution), pan-caspase inhibitor (Z-VAD-FMK), and a selective NLRP3 inflammasome inhibitor (MCC950), were adopted to elucidate the role of inflammasome activation in LCN2 secretion. As expected, the three inhibitors blocked IL-1β release in response to NG and MSU in the LPS-primed BMDM (Figure 2B). On the other hand, none of the inhibitors restored the suppressed LCN2 secretion by the NG or MSU treatments. Overall, the NLRP3 triggers repressed the secretion of LCN2 regardless of inflammasome activation.

### 3.3. NLRP3 Triggers Block LCN2 Transcription

The effects of an NLRP3 trigger on the transcription of the *Lcn2* gene were next confirmed. As shown in Figure 3A, NG was treated at different time points during the LPS priming step, and the *Lcn2* transcripts and proteins were then measured. NG significantly attenuated the transcription of the *Lcn2* gene (Figure 3B) and proteins (Supplementary Figure S1A). Various concentrations of NG were applied at the Ex1 of Figure 3A, and the transcripts of several cytokines were measured. As a result, the *Lcn2* transcripts (Figure 3C) and proteins (Supplementary Figure S1B) were diminished by the NG treatment in a dose-dependent manner. On the other hand, the expression of *pro-IL-1β*, *IL-6*, and *TNFα* mRNAs were not changed by NG (Figure 3C). The well-studied transcriptional regulator of the *Lcn2* gene [12], IκBζ (*Nfkbiz* gene), was also unchanged by the NG treatment (Figure 3C and Supplementary Figure S1C). The effects of NG on the *Lcn2* transcripts induced by the other TLR ligands or 12-O-tetradecanoylphorbol-13-acetate (TPA) [19] were also examined (Supplementary Figure S1D). As expected, the TLR signaling-mediated *Lcn2* transcription was blocked by the NG treatment. The effects of an NLRP3 trigger on the other cells expressing the *Lcn2* gene were examined further. RAW 264.7 cells, a mouse macrophage-like cell line, were treated with LPS and NG, as shown in Figure 3A, and the *Lcn2* mRNAs and proteins were then observed (Figure 3D and Supplementary Figure S1E). Similar to BMDM, NG attenuated LCN2 expression in RAW 264.7. The expression of *Lcn2* mRNA was analyzed further in peritoneal exudate cells (PECs) isolated from mice (Figure 2A). As shown in Supplementary Figure S1F, the NG injection attenuated the transcripts of *Lcn2* mRNA in PECs. Overall, NLRP3 triggers selectively inhibit the transcription of the *Lcn2* gene in mouse macrophages.

### 3.4. NLRP3 Triggers Inhibit LCN2 Production

**Figure 4:**
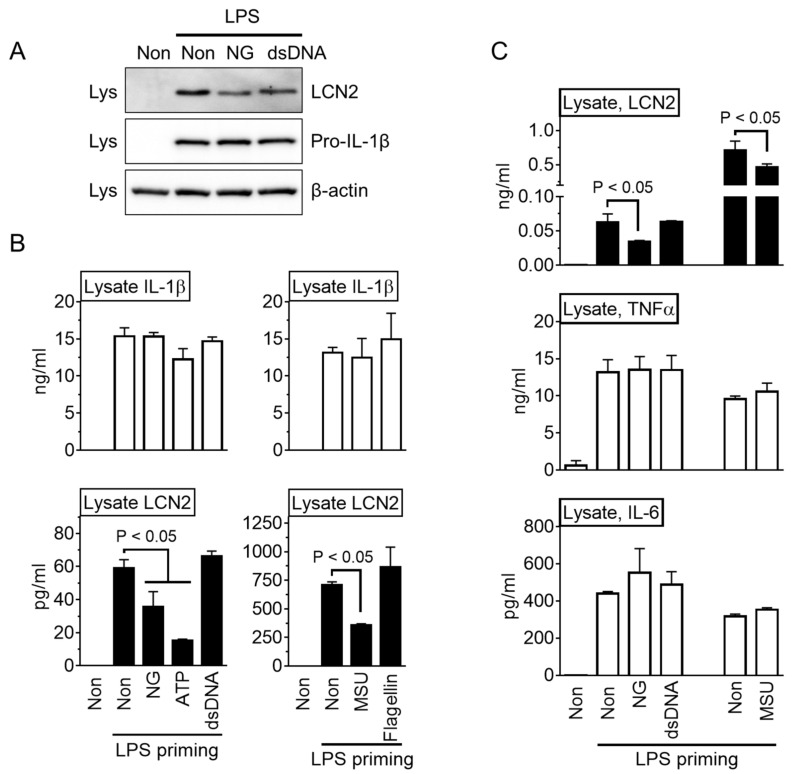
Effect of NLRP3 triggers on cytosolic LCN2 levels. (**A**) BMDM were primed with LPS and then treated with NG or dsDNA. The cellular lysates were subjected to immunoblotting as indicated. (**B**) LPS-primed BMDM were treated with an inflammasome trigger as indicated, and then the cellular lysates were analyzed by IL-1β or LCN2 ELISA. (**C**) LPS-primed BMDM were treated with inflammasome triggers as indicated, and the levels of IL-1β, TNFα, and IL-6 in the lysate were measured. The bar graph shows the mean ± SD of at least two independent experiments.

The cause of the reduced LCN2 secretion by NLRP3 triggers was determined by analyzing the cellular lysate of the macrophages. As a result (Figure 4A), the LCN2 proteins were reduced by the NG treatment but not by dsDNA transfection. In addition, the intracellular level of the LCN2 protein was measured in the same samples of Figure 1C. As shown in Figure 4B, the LCN2 levels in the lysate were attenuated significantly by the NLRP3 trigger (NG, ATP, and MSU) while the cytosolic IL-1β proteins were not. Furthermore, the effects of NLRP3 triggers on the TNFα and IL-6 levels in the lysates were determined. As shown in Figure 4C, NLRP3 triggers only diminished the level of LCN2 but not TNFα and IL-6. Therefore, NLRP3 triggers inhibited LCN2 secretion through the suppression of LCN2 production.

## 4. Discussion

In this study, the activation of inflammasome regulated the secretion of LCN2 in mouse macrophages, and the NLRP3 triggers attenuated LCN2 expression. When mouse BMDM were activated by NLRP3 inflammasome, IL-1β secretion was inversely proportional to LCN2 release. On the other hand, the change in LCN2 secretion was not observed in the BMDM activated with NLRC4 or AIM2 triggers. Similar to the in vitro results, LCN2 secretion from the LPS-injected mice was attenuated by the NG treatment, an NLRP3 trigger. A pharmacological inhibitor of inflammasome activation could not restore the decrease in LCN2 secretion by the NLRP3 triggers, suggesting that the decrease in LCN2 was independent of inflammasome activation. To determine why the NLRP3 triggers inhibit LCN2 secretion, this study analyzed the cytosol LCN2 proteins and *Lcn2* transcripts in macrophages treated with LPS and NG, an exogenous NLRP3 trigger without stimulating TLR signaling. As a result, NG attenuated not only the cytosolic LCN2 proteins but also *Lcn2* transcription. On the other hand, NG did not alter the cytokine expression in response to an LPS treatment. Overall, NLRP3 triggers selectively attenuate the TLRs-mediated LCN2 expression in mouse macrophages.

LCN2 is expressed in various cells, including cancer cells, and its expressing pattern varies. Based on the promoter study of the *Lcn2* gene, several transcriptional factors (e.g., NF-κB, C/EBPβ, CREB, STAT1, and STAT3) have been determined, suggesting that LCN2 is involved in inflammation and metabolism [6]. In human macrophages, *Lcn2* gene expression was induced by STAT3 and C/EBPβ [20]. In addition, STAT1 and NF-κB are mediated to induce *Lcn2* transcripts in adipocytes [21]. Some human cancers strongly express LCN2 associated with the tumor size and invasiveness [22]. The *Lcn2* gene promoter of the esophageal squamous cell carcinoma possesses a TPA-response element that interacts with several transcriptional factors to induce *Lcn2* transcripts [22]. TPA is one of the inducers of LCN2 expression through the MEK-ERK signal pathway [19]. In the current study, *Lcn2* mRNA of BMDM was upregulated by TPA, and *Lcn2* expression was attenuated by the NG treatment (Supplementary Figure S1D). Furthermore, this study examined whether NLRP3 triggers stimulated the protein lysis pathways through autophagy, lysosome, or proteasome. As shown in Supplementary Figure S2, the LPS-primed BMDMs were treated with the following inhibitors: chloroquine diphosphate, MG-132, 3-methyladenine, and ammonium chloride. As a result, no single inhibitor restored the attenuated LCN2 secretion by ATP. Overall, NLRP3 triggers may block at least one of the transcriptional signal pathways of the *Lcn2* gene promoter.

The lung epithelial cell line, A549, induces LCN2 synthesis by an IL-1β treatment but not by TNFα, even though the two cytokines mediate the interaction of NF-κB on its binding site of the *Lcn2* promoter [12]. This difference is explained by IκBζ (*Nfkbiz* gene), an atypical inhibitor of κB and a coactivator of the NF-κB target gene, that is induced by IL-1β not by TNFα [12,23]. TNFα also induces the expression of the *Lcn2* gene if the cells are co-stimulated with IL-17, which stabilizes the *Nfkbiz* gene and accumulates the IκBζ protein [24]. The importance of IκBζ on *Lcn2* expression was also confirmed using IκBζ-deficient macrophages (Supplementary Figure S1G). Based on these findings, it was hypothesized that NLRP3 triggers down-regulated *Lcn2* expression by inhibiting *Nfkbiz* expression (Figure 3C). On the other hand, NG did not alter the expression of the *Nfkbiz* gene. Based on the literature, the common feature of NLRP3 triggers (i.e., ATP, MSU, and NG) is that they activate the NLRP3 inflammasome through potassium efflux [10]. NG, an exogenous molecule unlike ATP and MSU, is an antibiotic potassium ionophore that enhances the cytotoxic effect, suggesting that it is an anti-cancer drug [25,26]. The biological function of NG has not been well studied. Although the precise role of the inhibition of *Lcn2* transcription was not presented, it suggests that LCN2 secretion decreased during NLRP3 inflammasome activation.

LCN2 secretion is induced by infection, damage, and metabolic disorders in various tissues and organs [2]. In contrast, LCN2 has been suggested to be an anti-inflammatory regulator [6]. LCN2 secretion from mice suffering from pneumococcal pneumonia inhibited the early phase of inflammation, leading to increased mortality [27]. LPS produces LCN2 in macrophages, but recombinant LCN2 blocks proinflammatory cytokine synthesis [28]. Furthermore, LCN2 regulates macrophage polarization to M2 type which attenuates inflammation [6]. As stated above, the role of LCN2 on inflammation is controversial. Therefore, the physiological meaning of the regulation of LCN2 by NLRP3 triggers is complicated. This report suggested that NLRP3 triggers activated inflammasome and attenuated the expression of LCN2, another component of the innate immunity.

## Data Availability

Not applicable.

## Supplementary Materials

The following are available online at https://www.mdpi.com/article/10.3390/cells10071660/s1, Figure S1: Effect of NLRP3 trigger on LCN2 secretion, Figure S2: Effect of the protein lysis pathways on LCN2 secretion, Table S1: Primers sequences.
