# Supplementary material for: NLRP3 Triggers Attenuate Lipocalin-2 Expression Independent with Inflammasome Activation
Source: Cells. 2021 Jul 2;10(7):1660. doi: 10.3390/cells10071660 (PMC8305203; doi:10.3390/cells10071660)

Supplementary Information

NLRP3 triggers attenuate lipocalin-2 expression independent with inflammasome activation

Huijeong Ahn[1#], Gilyoung Lee[1#], Jeongeun Kim[1#], Jeongho Park[1], Seung Goo Kang[2], Sung-Il Yoon[2], Eunsong Lee[1] and Geun-Shik Lee[1]*

[1]College of Veterinary Medicine and Institute of Veterinary Science, Kangwon National University, Chuncheon, Gangwon, 24341, Korea.

[2]Division of Biomedical Convergence, College of Biomedical Science, Kangwon National University, Chuncheon, Gangwon, 24341, Korea.

[#], These authors contributed equally to this work.

*Correspondence: Geun-Shik Lee, D. V. M., Ph. D.

Laboratory of Inflammatory Diseases, Department of Physiology, College of Veterinary Medicine, Kangwon National University, Chuncheon, Gangwon, 24341, Republic of Korea.

e-mail: leegeun@kangwon.ac.kr, Tel: +82-33-250-8683, Fax: +82-33-244-2367

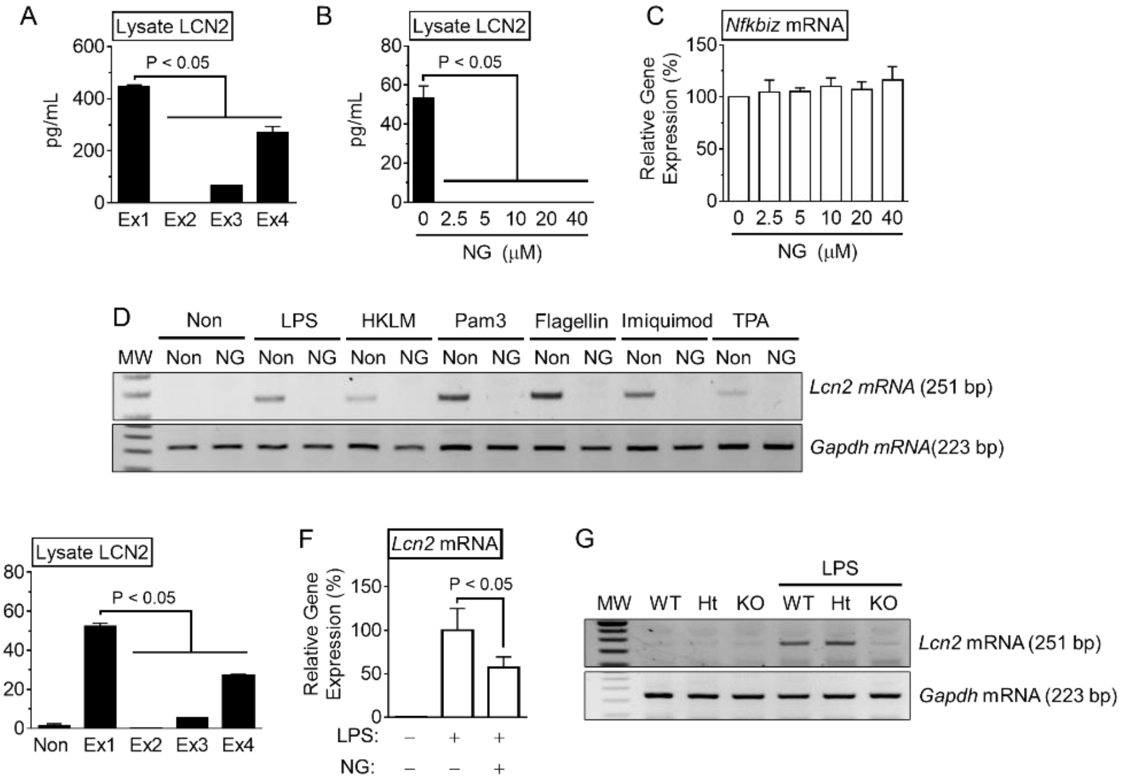

Figure S1. Effect of NLRP3 trigger on LCN2 secretion. **A**, BMDM were treated with LPS and NG, as indicated in Fig. 4A. The cytosolic LCN2 protein was measured by ELISA. **B**, BMDM were treated with LPS and various dosages of NG as indicated in Ex1 of Fig. 4A. The intracellular LCN2 protein was analyzed by ELISA. **C**, The band density of *Nfkbiz* mRNA of Fig. 4C was analzyed and is presented as a bar graph. **D**, BMDM were treated with LPS (10 ng/ml), heat-killed *Listeria monocytogens* (HKLM, 1%), Pam3CSK (0.1 μ g/ml), flagellin (500 ng/ml), imiquimod (5 μg/ml), 12-O-tetradecanoylphorbol-13-acetate (TPA, 10 μg/ml) for 3h in the presence of NG, as indicated in Ex1 of Fig. 4A. *Lcn2* mRNA expression was analyzed by RT-PCR. **E**, Raw 264.7 cells were treated with LPS and NG as indicated in Fig. 4A. The cytosolic LCN2 was analyzed by ELISA. **F**, The peritoneal exudate cells (PECs) were harvested from the mice of Fig. 2A. *Lcn2* mRNA expression of PECs was analyzed by real-time qPCR. **G**, BMDM derived from *Nfkbiz*$^{+/+}$ (WT), *Nfkbiz*$^{+/-}$ (HT), or *Nfkbiz*$^{-/-}$ (KO) mice [Ref] were treated with/without LPS (10 ng/ml) for 3h. *Lcn2* mRNA expression was measured by RT-PCR. The bar graph shows the mean ± SD.

[Ref] J. Kim, H. Ahn, S. Yu, J.H. Ahn, H.J. Ko, M.N. Kweon, E.J. Hong, B.S. An, E. Lee, G.S. Lee, IkappaBzeta controls NLRP3 inflammasome activation via upregulation of the Nlrp3 gene, Cytokine 127 (2020) 154983.

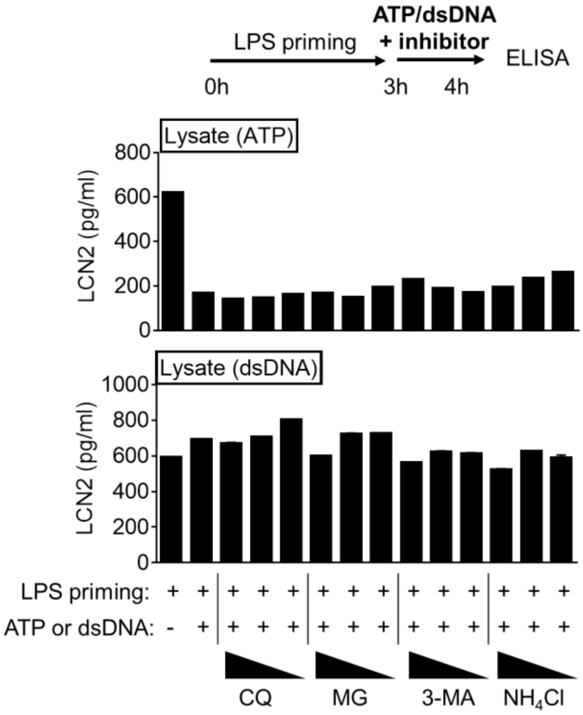

Figure S2. Effect of the protein lysis pathways on LCN2 secretion. BMDMs were treated with LPS, and then treatd with ATP or dsDNA in the presence of the flowing inhibitors: chloroquine diphosphate (CQ; 5, 25, and 50 μg/mL; an autophagy inhibitor), MG-132 (MG; 0.5, 2.5, and 5 μM; a proteasome inhibitor), 3-methyladenine (3-MA; 0.5, 2.5, and 5 μM; an autophagy inhibitor), and ammonium chloride (NH_4Cl; 2, 10, and 20 mM; a lysosome inhibitor). The secretion of LCN2 were measured by ELISA.

Table S1. Primers sequences.

Gene	GenBank ID	Sequence (5’ to 3’)I	Size (bp)
Lcn2	NM_008491	GGC CAG TTC ACT CTG GGA AA	251
		TCG GTG GGG ACA GAG AAG AT	
Pro-IL-1β	NM_008361	CAG GCA GGC AGT ATC ACT CA	350
		AGG CCA CAG GTA TTT TGT CG	
IL-6	NM_031168	GTT CTC TGG GAA ATC GTG GA	339
		GGA AAT TGG GGT AGG AAG GA	
TNFα	NM_013693	ACG GCA TGG ATC TCA AAG AC	324
		CGG ACT CCG CAA AGT CTA AG	
Nfkbiz	NM_001159394	GTG TTC GCG TGA AGA ACT CG	208
		AGA GCT GGC CTC TTG CAA AC	
Gapdh	NM_013693	AAC TTT GGC ATT GTG GAA GG	223
		ACA CAT TGG GGG TAG GAA CA	

Original Images for Blots of Fig. 3A

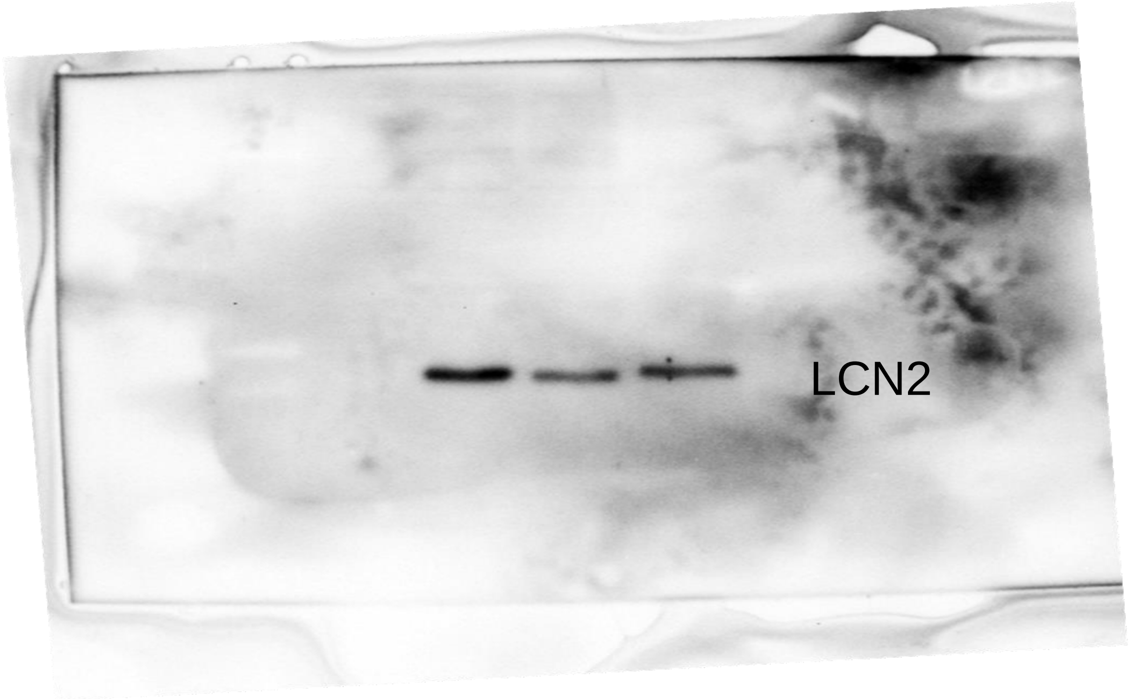

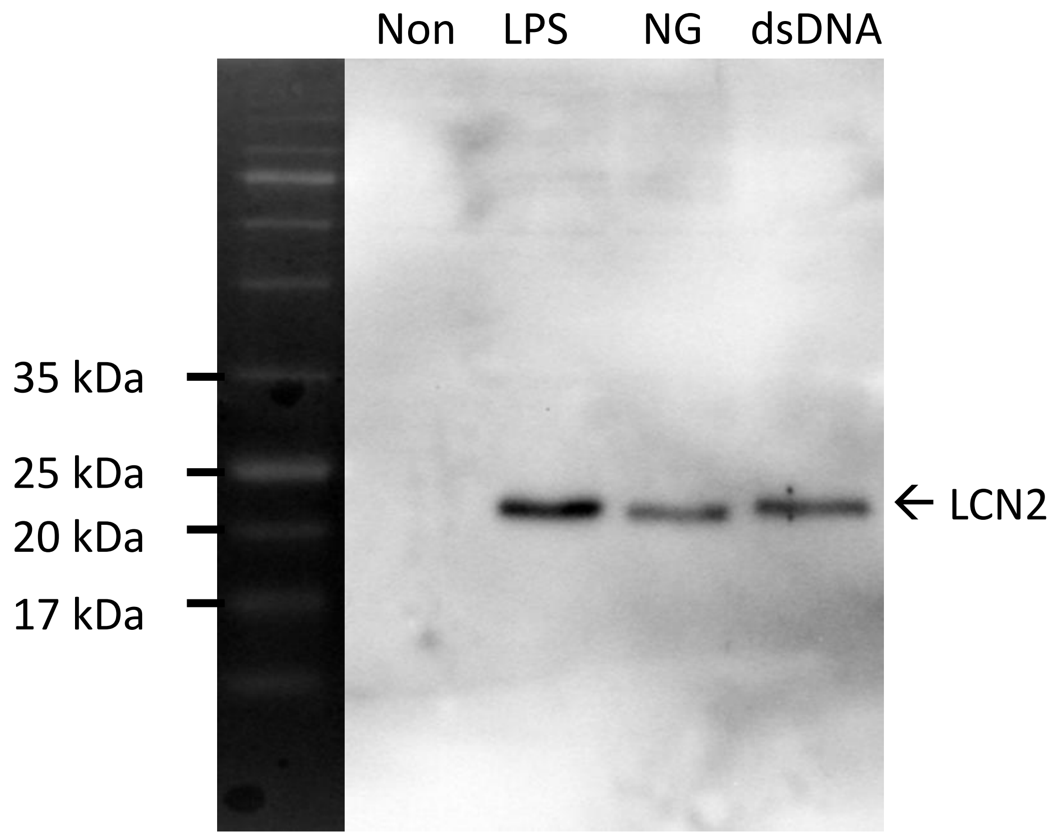

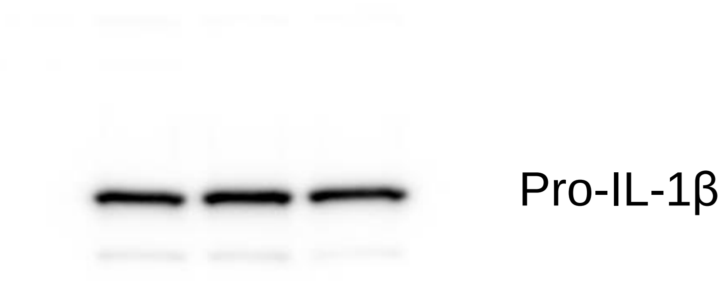

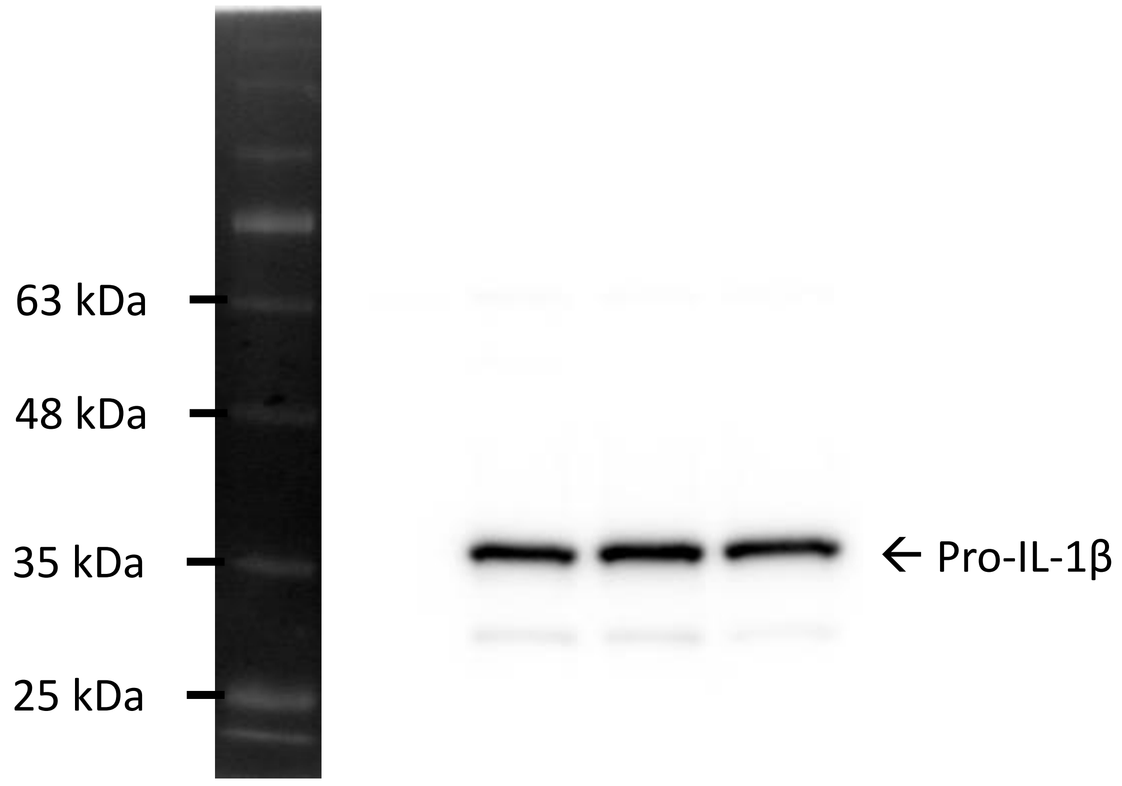

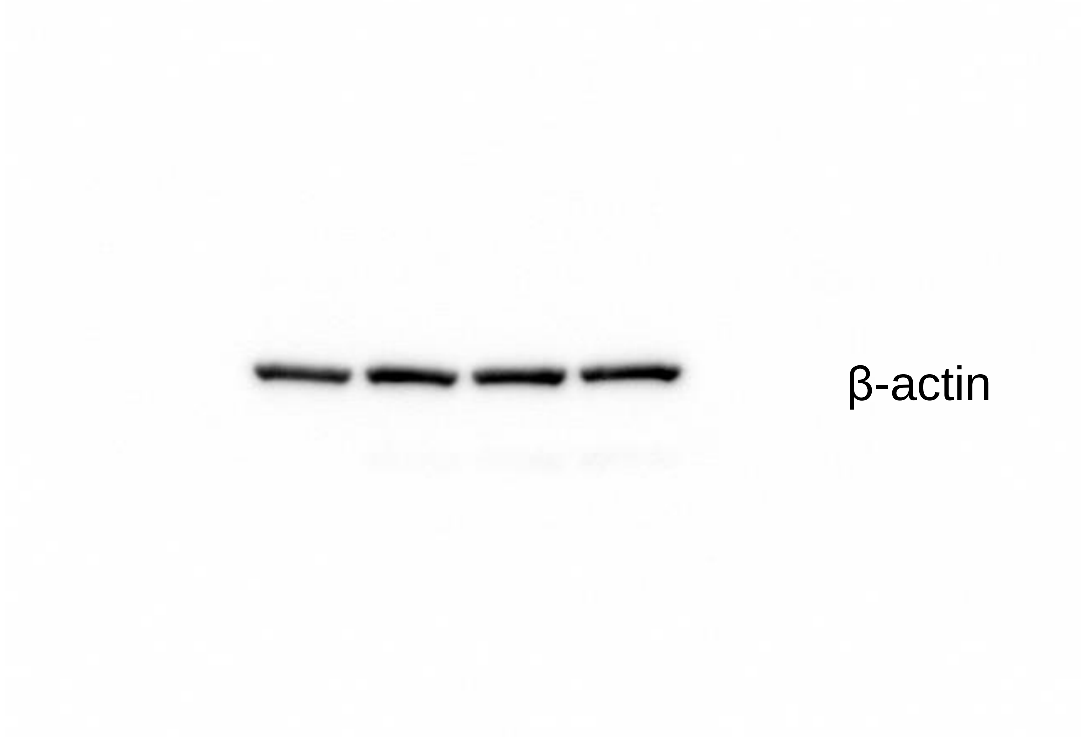

Original Images for Gels of Fig. 4B

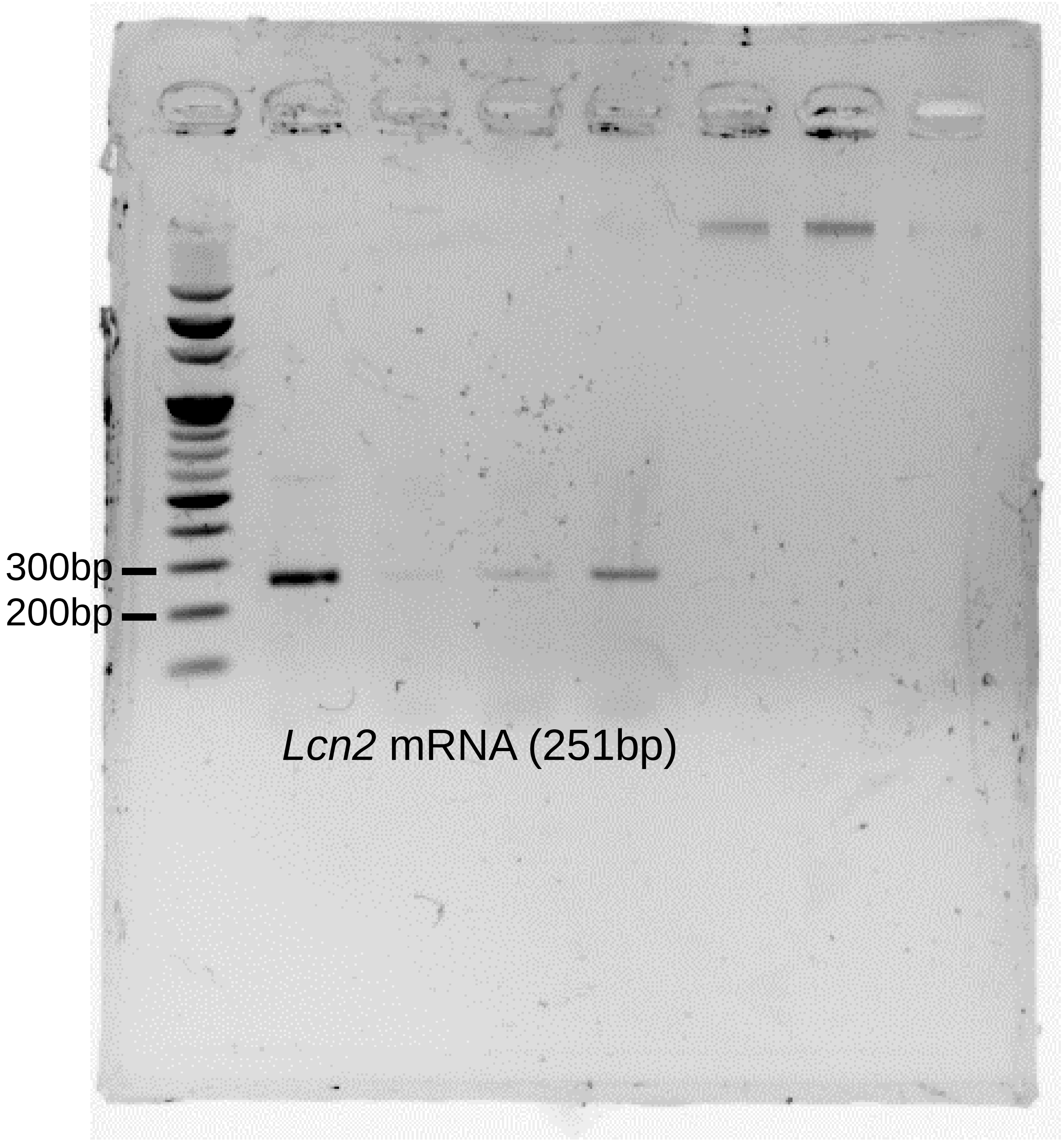

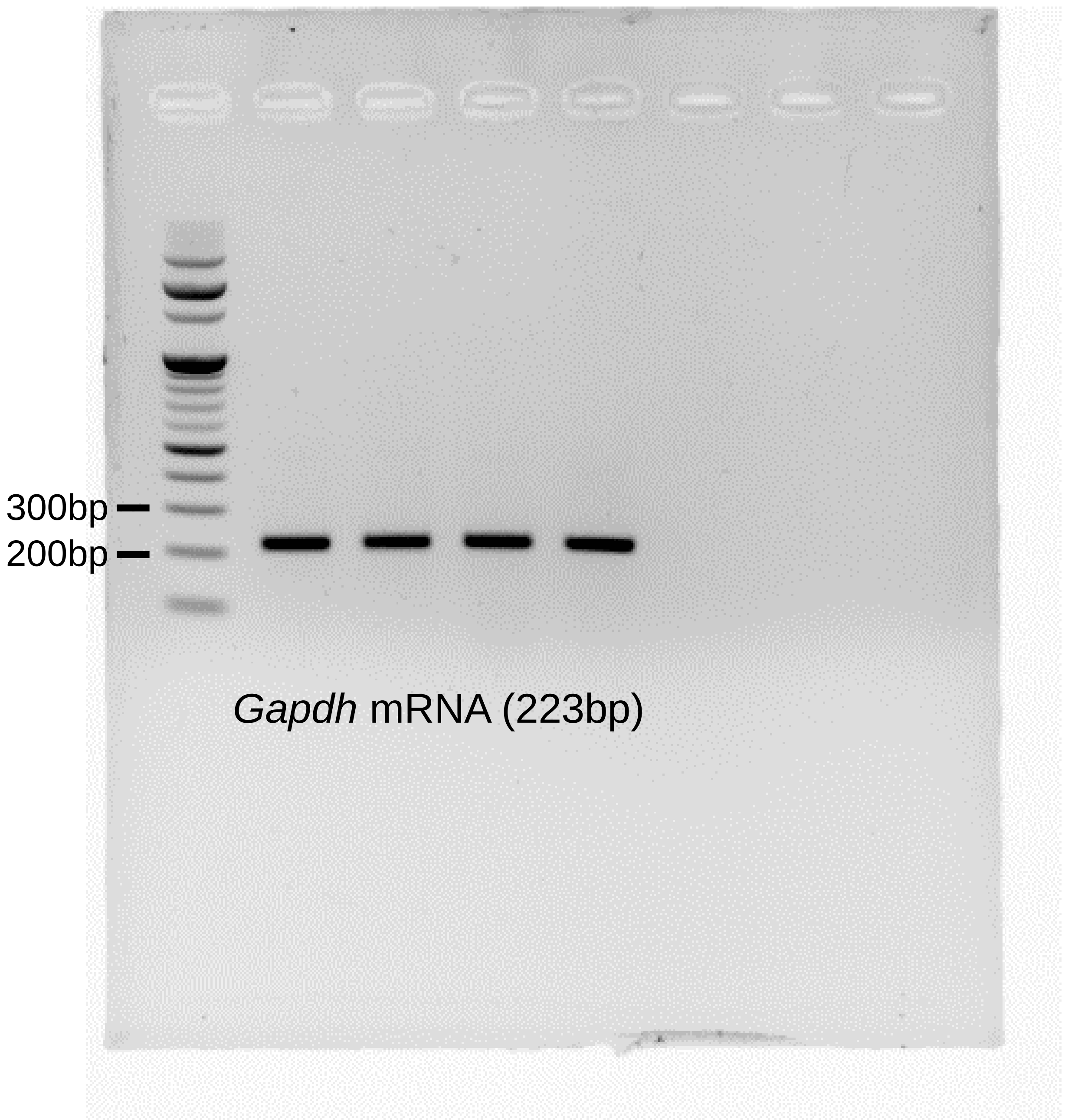

Original Images for Gels of Fig. 4C

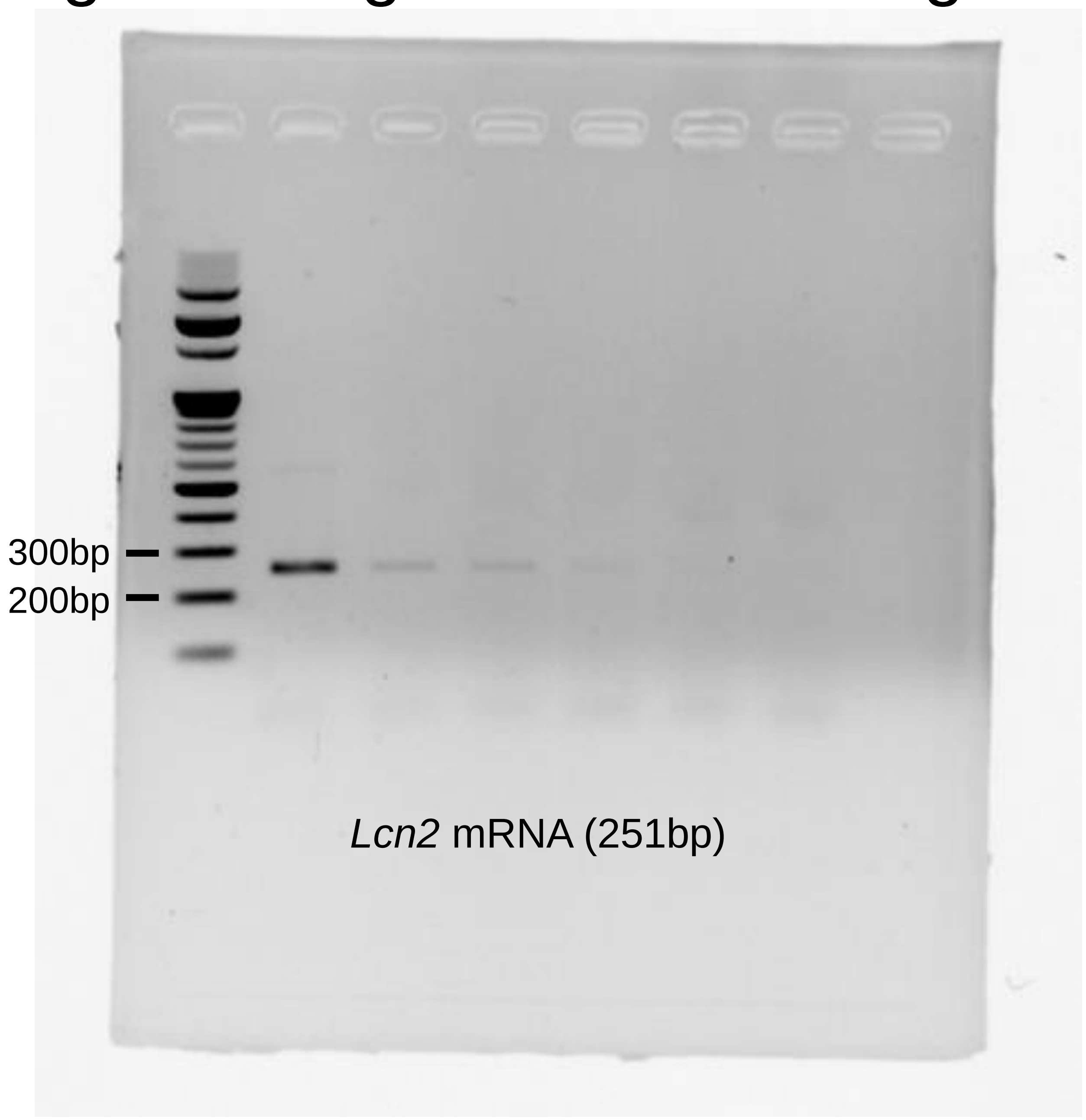

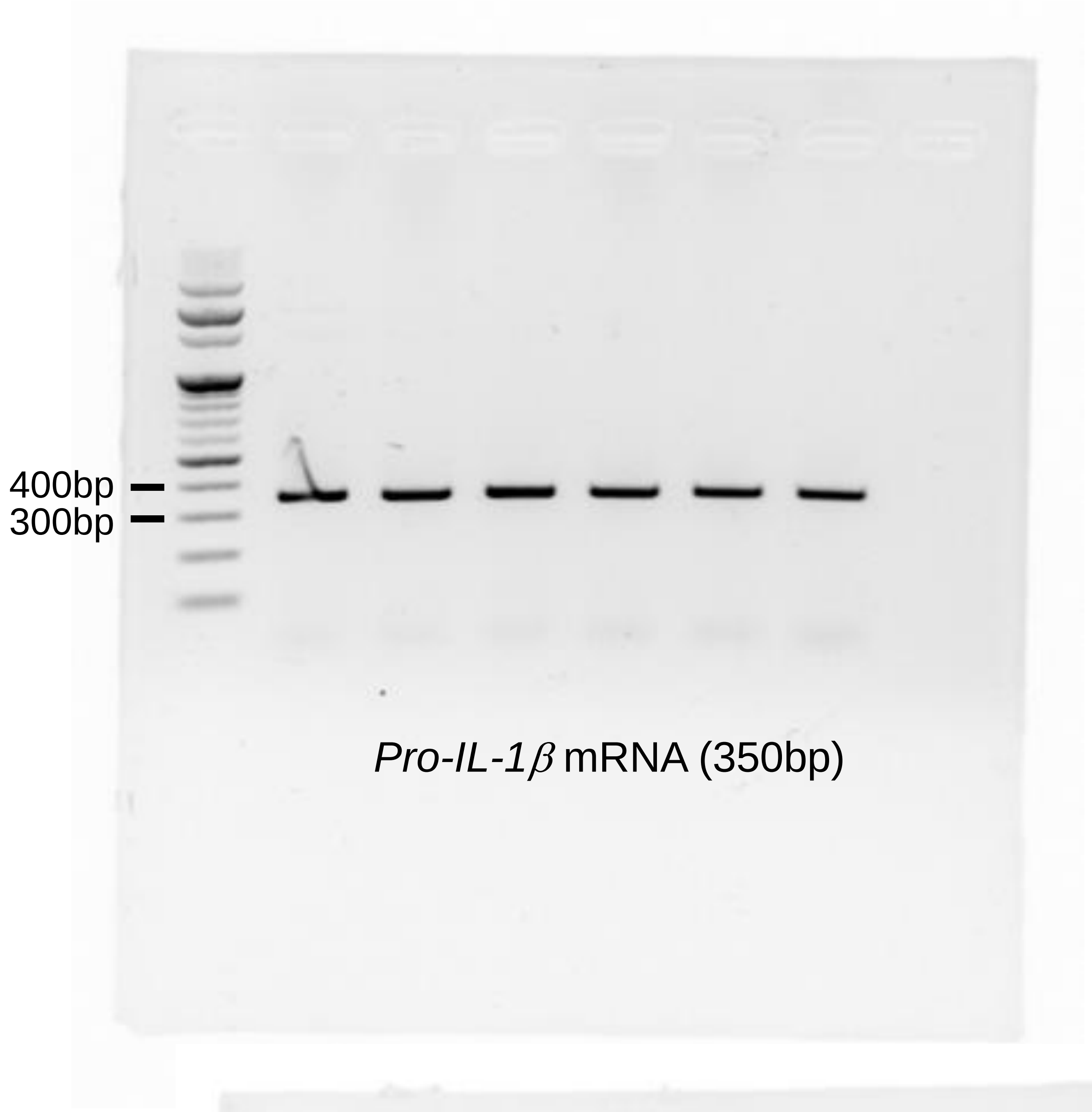

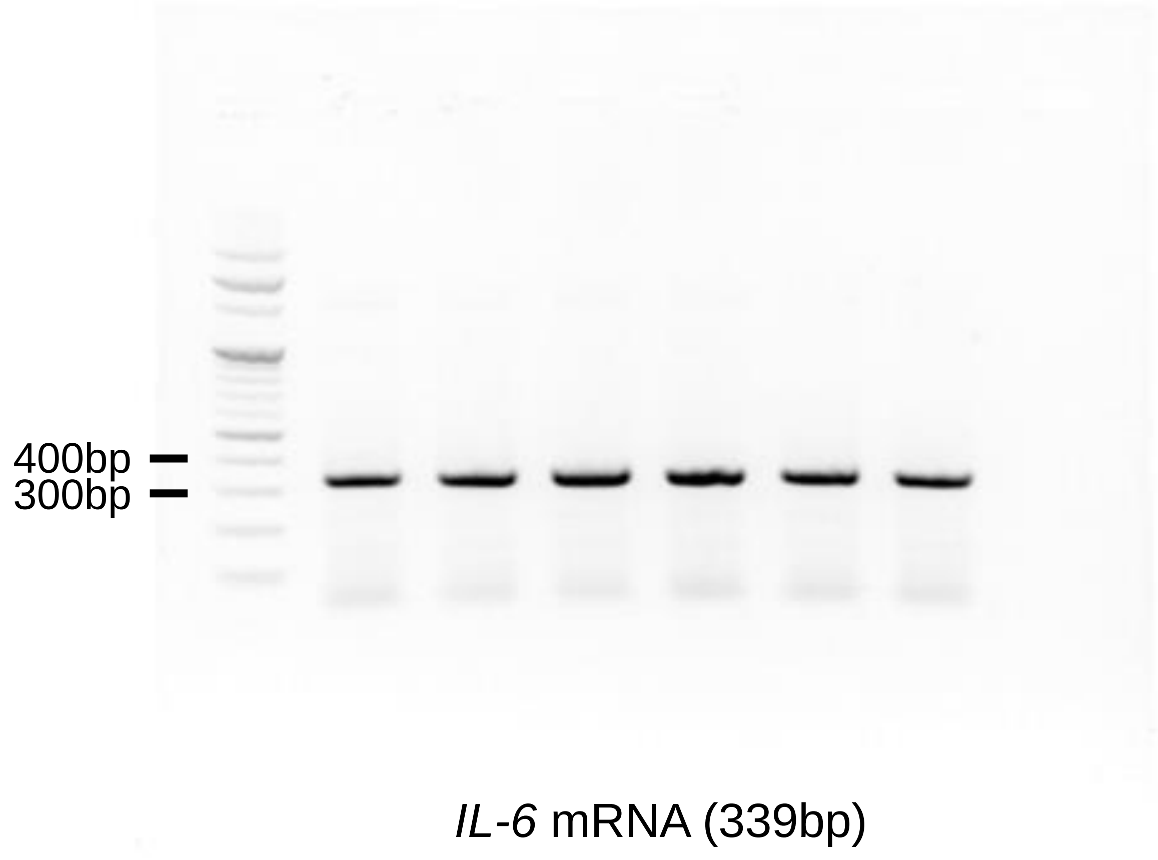

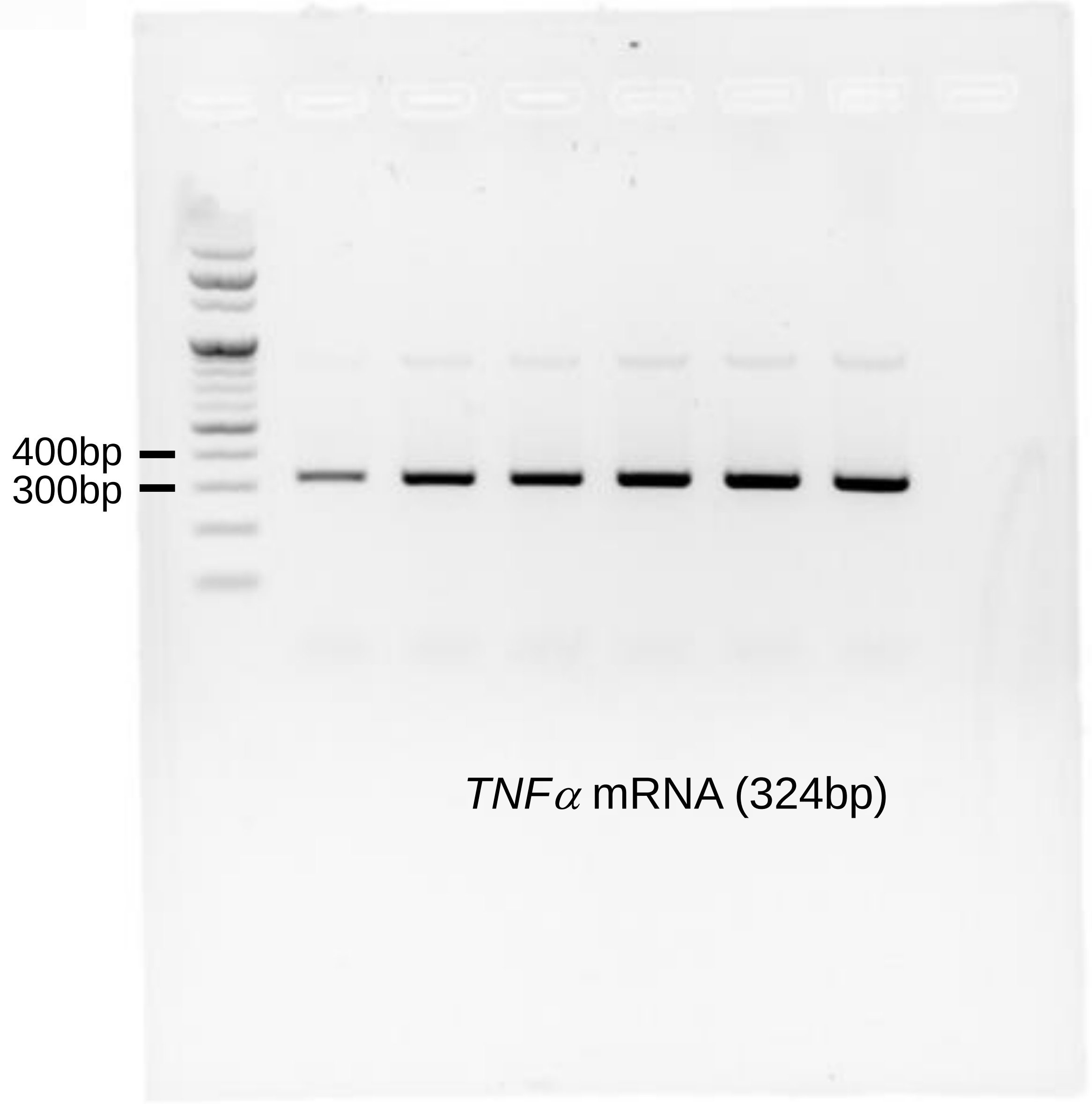

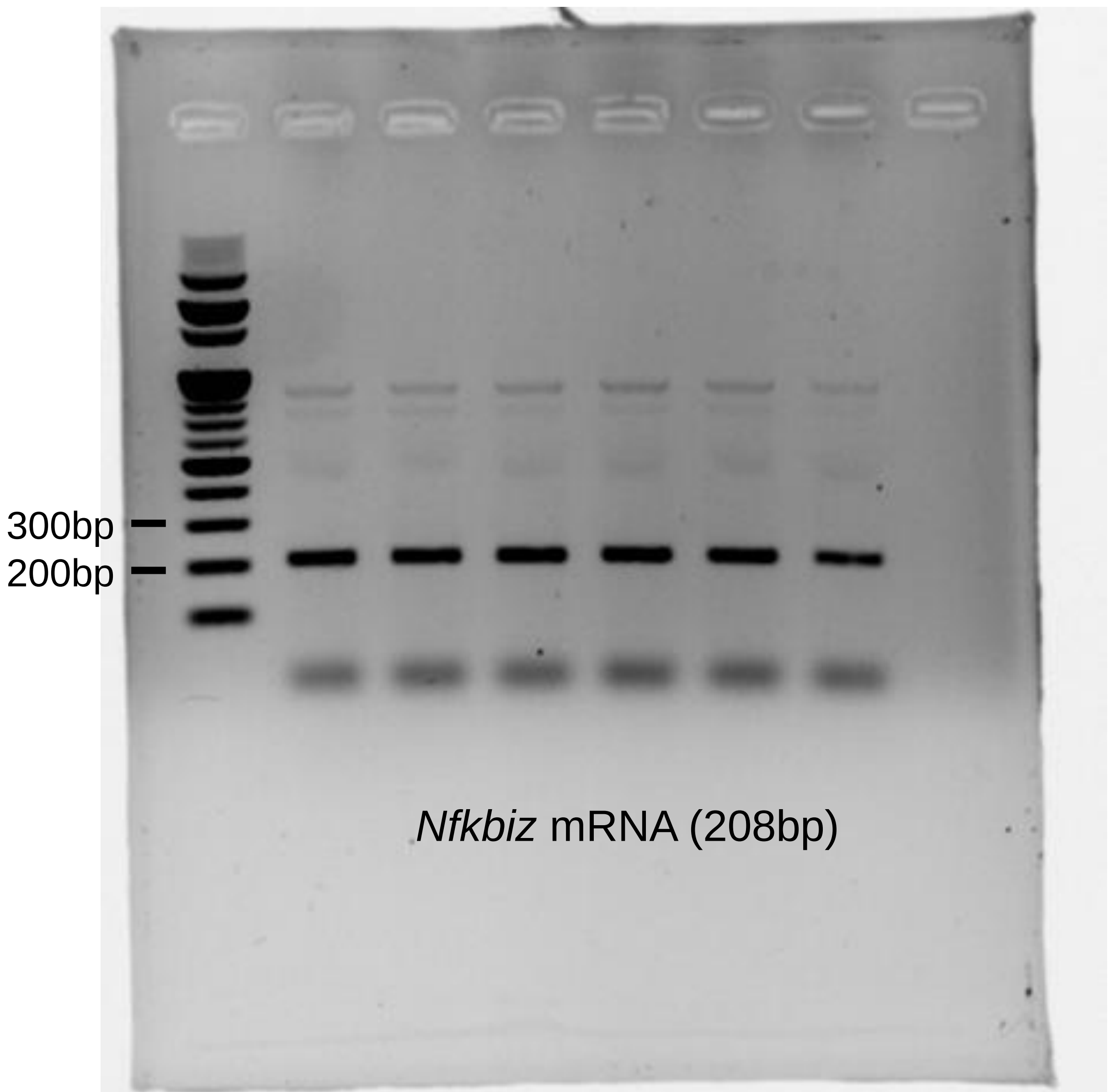

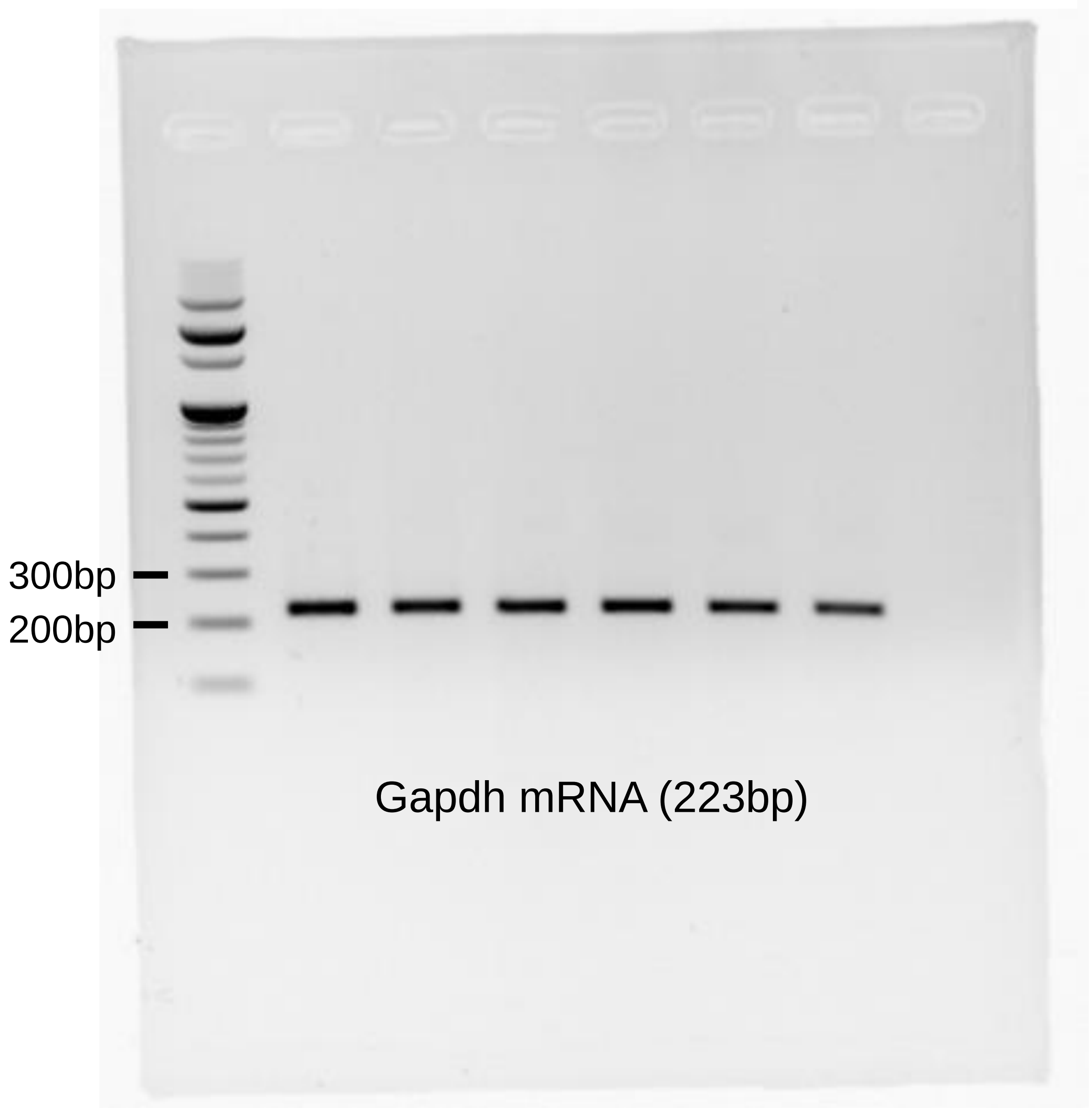

Original Images for Gels of Fig. 4D

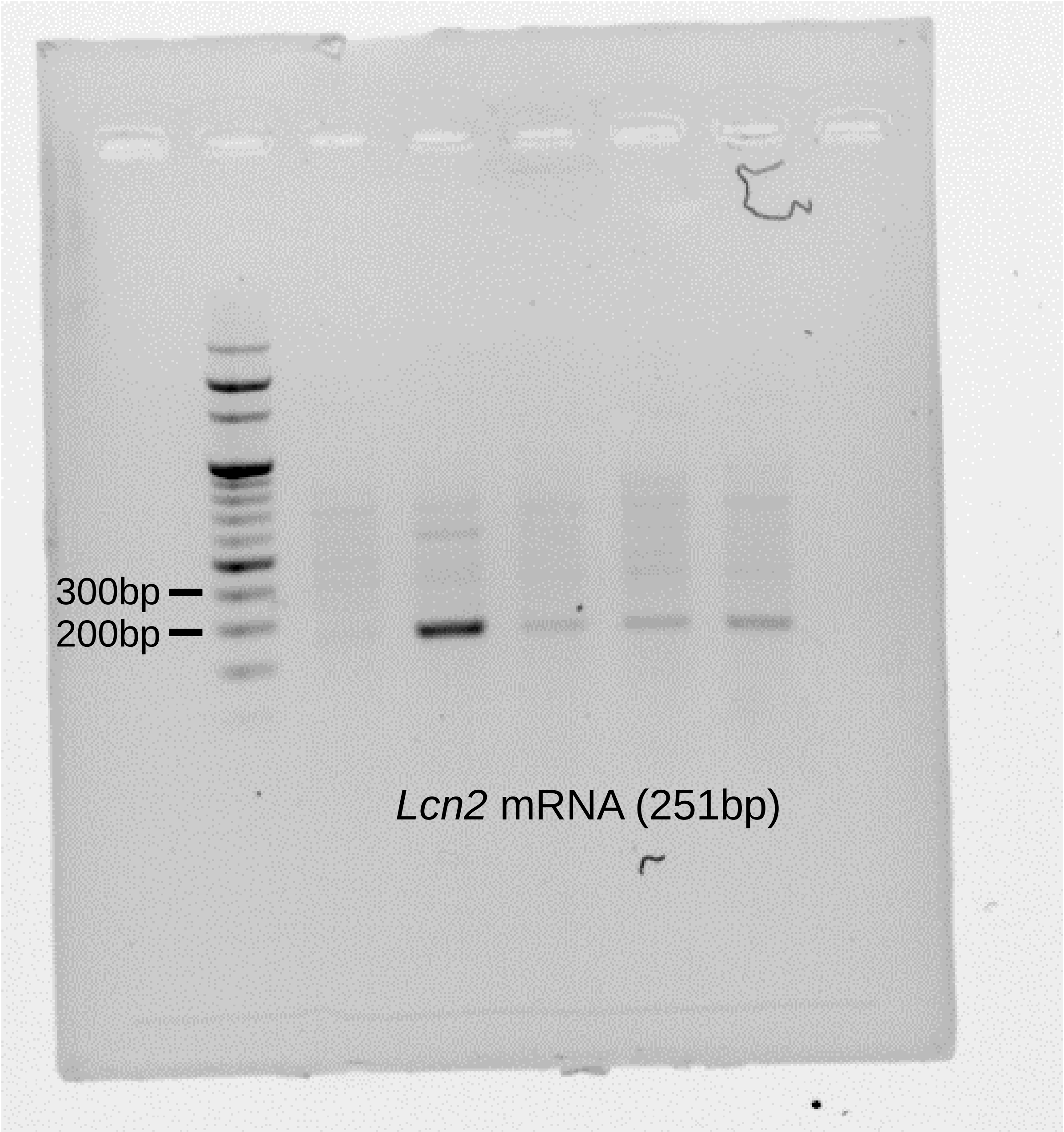

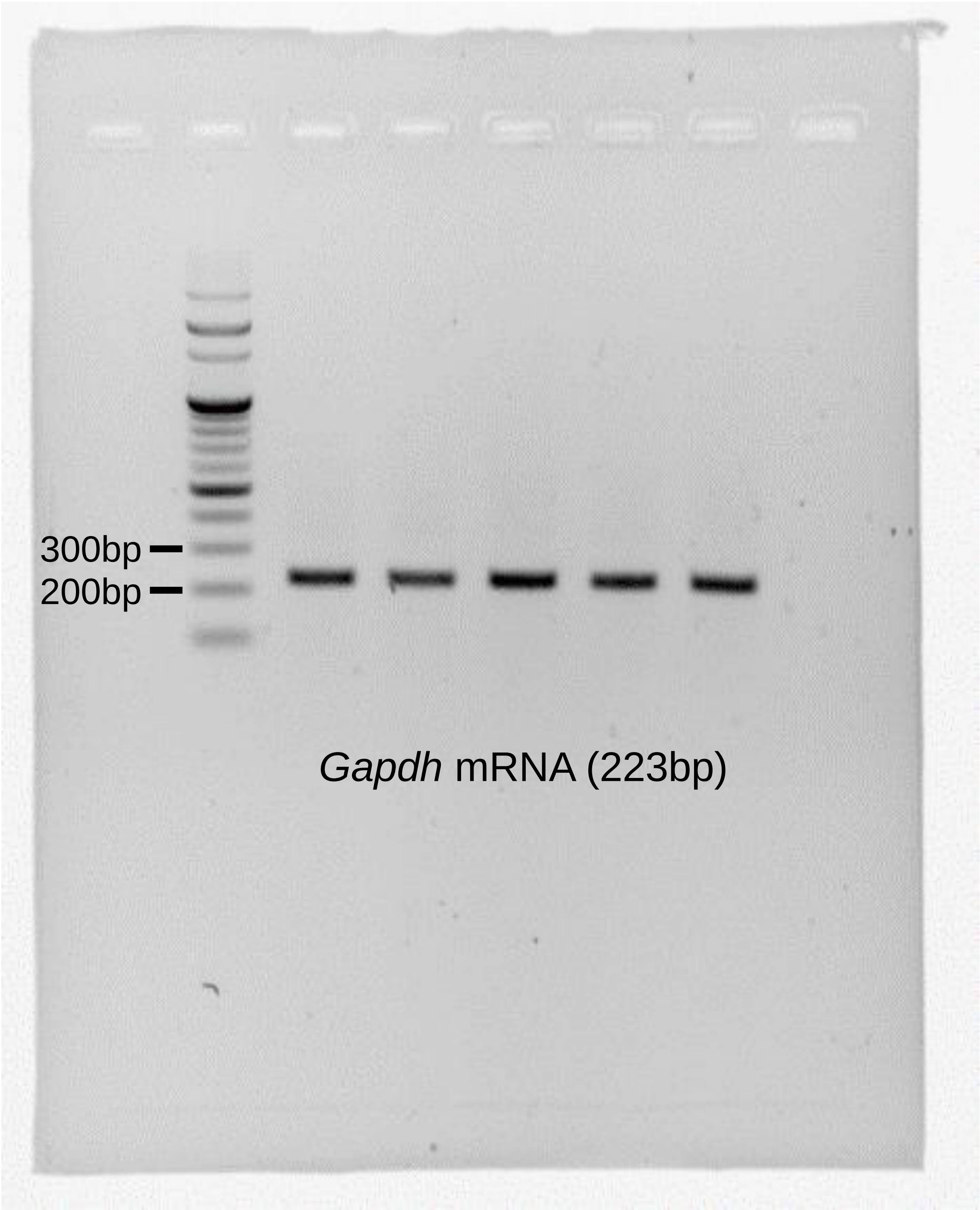

Supplement: Supplementary file 1 [file cells-10-01660-s001.zip › cells-1227527-supplementary.pdf]
